# Congenital and Acquired Abnormalities of the Corpus Callosum: A Pictorial Essay

**DOI:** 10.1155/2013/265619

**Published:** 2013-08-06

**Authors:** Katarzyna Krupa, Monika Bekiesinska-Figatowska

**Affiliations:** Department of Diagnostic Imaging, Institute of Mother and Child, ul. Kasprzaka, 17a, 01-211 Warsaw, Poland

## Abstract

The purpose of this review is to illustrate the wide spectrum of lesions in the corpus callosum, both congenital and acquired: developmental abnormalities, phakomatoses, neurometabolic disorders, demyelinating diseases, infection and inflammation, vascular lesions, neoplasms, traumatic and iatrogenic injury, and others. Cases include fetuses, children, and adults with rich iconography from the authors' own archive.

## 1. Introduction

Corpus callosum is one of the three interhemispheric commissures (anterior commissure, hippocampal commissure and corpus callosum) and the greatest of them—it consists of approximately 190 000 000 axons [1]. Its role is interhemispheric connection and coordination. A good example of this role is an alien hand syndrome (AHS): anterior callosal injury (in case of stroke, trauma, and tumor) leads to intermanual conflict with involuntary movements of nondominant hand. The callosal type of AHS can best be explained by the loss of interhemispheric connection, revealed during activities that require control of the dominant hemisphere [2]. Another example is the role of the corpus callosum in the interhemispheric spread of epileptic activity and the efficacy of corpus callosotomy in cases of medically intractable epilepsy [3]. The patients that underwent callosotomy present a “disconnection syndrome” as well as subtle social and emotional deficits [1]. Transection of the anterior part of the callosal body during neurosurgical procedures aiming at the removal of tumors of the third or lateral ventricles leads to the deficits of memory, the dysexecutive cognitive and behavioral syndrome, disturbances in interhemispheric transfer of learning from one hand to the other, and an increase in reaction times [4].

In the anterior-posterior direction the corpus callosum is divided into rostrum, genu, body, isthmus, and splenium. The fibers in the rostrum connect fronto-basal cortex, in the genu—prefrontal cortex and anterior cingulate area, in the body—precentral (motor) cortex, insula, and cingulate gyri, in the isthmus—precentral and postcentral gyri (motor, somatosensory) and primary auditory areas, and in the splenium—posterior parietal, medial occipital, and medial temporal cortexes [5].

According to the newest theory the corpus callosum in its embryological development is fused of two separate parts: the anterior one, consisting of the rostrum, genu, and body and the posterior one—splenium. The place of the fusion is the isthmus. Callosal development is a very quick process and takes place in 13th week of gestational life. From this time on the corpus callosum grows mainly in the anterior direction, pushing the splenium posteriorly. It reaches its final shape in midgestation (week 20) but is still small and grows, initially by addition of fibers and later by myelination [5]. The target volume is reached at the age of 6–9 years.

Myelination of the brain progresses from the center to the periphery, from bottom to top and from back to front. In newborns corpus callosum is not yet myelinated. In the 6th month after birth, when the cerebellum and genu of the internal capsule completed the process of myelination, the corpus callosum is myelinated in part (splenium), although it has not yet reached its target volume. Callosal genu is myelinated a little bit later than splenium—in about 8th month. It is not until about the first year of life that the corpus callosum displays the typical signal intensity: hyperintense on T1-weighted images and hypointense on T2-weighted images.

There are a few papers illustrating callosal pathology in the literature [6–8]. Continuing our work from the past [9] we present a more detailed review of congenital and acquired callosal changes in fetuses, children, and adults with rich iconography from the own archive.

## 2. Acquisition Parameters of Presented Images

All the images were acquired with 1.5T scanners. Figures 9, 12, 21, 24, and 25 were obtained with Philips Gyroscan ACS-NT in the years 1999–2006. Figures 4, 18, 20, 22, 23, 28, 29, 30, and 34 (in adult patients) were acquired with one GE Signa HDxt scanner in the years 2008–2013 and the remaining figures (in children) were acquired with another GE Signa HDxt scanner in the years 2004–2013.

The following acquisition parameters were used:

- Philips Gyroscan ACS-NT
  - SE/T1-weighted images (T1WI): repetition time TR shortest, echo time TE 14 ms, flip angle FA 90 deg, number of acquisitions NEX 2, matrix MX 256 × 225, field of view FOV 25 cm, slice thickness/interslice gap ST 5.0/1.0 mm,
  - TSE/T2WI: TR shortest, TE 100 ms, FA 90 deg, NEX 2, MX 512 × 512, FOV 25 cm, ST 5.0/1.0 mm,
  - FLAIR: TR 6000 ms, TE 100 ms, FA 90 deg, NEX 2, MX 256 × 256, FOV 25 cm, ST 5.0/1.0 mm,
  - FFE/T2∗WI: TR shortest, TE 23 ms, FA 15 deg, NEX 2, MX 256 × 256, FOV 23 cm, ST 5.0/1.0 mm,
  - DWI: TR shortest, TE 42 ms, FA 90, NEX 1, ST 6.0/1.0 mm.
- GE Signa HDxt (adult brain)
  - SE/T1WI: TR 540 ms, TE 9 ms, NEX 2, MX 320 × 224, FOV 24 × 18 cm, ST 5.0/1.5 mm,
  - FSE/T2WI: TR 5000 ms, TE 88 ms, NEX 1.5, MX 384 × 384, FOV 24 × 24 cm, ST 5.0/1.5 mm,
  - FLAIR: TR 8002 ms, TE 126.8 ms, TI 2000, NEX 1, MX 320 × 256, FOV 24 × 24 cm, ST 5.0/1.5 mm,
  - GRE/T2∗WI: TR 660 ms, TE 15 ms, NEX 2, MX 320 × 192, FOV 24 × 18 cm, ST 5.0/1.5 mm,
  - DWI: TR 8000 ms, TE 98.5 ms, NEX 1, MX 128 × 128, ST 5.0/0.0 mm, *b* = 1000.
- GE Signa HDxt (children)
  - SE/T1WI: TR 540 ms, TE 9 ms, NEX 2, MX 320 × 224, FOV 24 × 18 cm, ST 5.0/1.5 mm,
  - FSE/T2WI: TR 5500 ms, TE 84 ms, NEX 1.5, MX 320 × 320, FOV 24 × 24 cm, ST 5.0/1.5 mm,
  - FLAIR: TR 8000 ms, TE 140.2 ms, TI 2000, NEX 1, MX 320 × 320, FOV 24 × 18 cm, ST 5.0/1.5 mm,
  - GRE/T2∗WI: TR 660 ms, TE 15 ms, NEX 2, MX 320 × 192, FOV 24 × 18 cm, ST 5.0/1.5 mm,
  - SWI: TR 6750 ms, TE 40 ms, NEX 4, MX 256 × 512, FOV 26 × 26 cm, ST 3.0/0.0 mm,
  - DWI: TR 6625 ms, TE 100.5 ms, NEX 2, MX 160 × 160, ST 5.0/1.5 mm, *b* = 1000,
  - FSPGR/3D/T1WI: TR 8.1 ms, TE 3.6 ms, TI 450, NEX 1, MX 320 × 224, FOV 24 × 24 cm, ST 1.6/−0.8 mm,
  - CUBE/3D/FLAIR: TR 6000 ms, TE 130.7 ms, TI 1852, NEX 1, MX 224 × 224, FOV 22 × 22 cm, ST 1.4/−0.7 mm.

## 3. Developmental Abnormalities

### 3.1. Agenesis and Hypoplasia

Incomplete or abnormal development leads to the most common pathology, which affects the organs in question: agenesis and hypoplasia.

**Figure 1:**
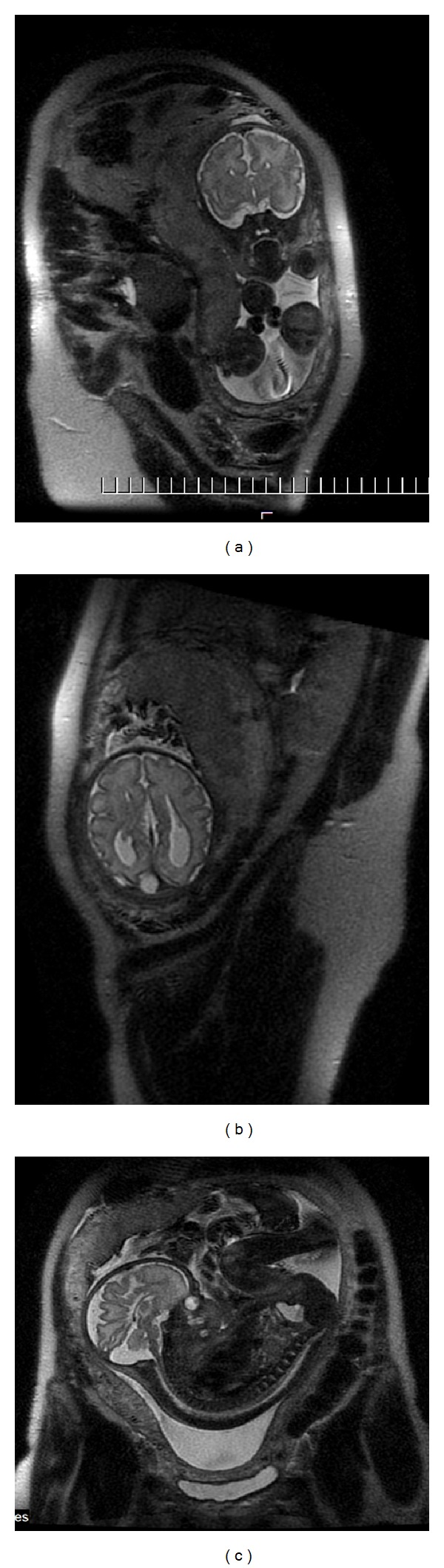
MRI of fetal brain. SSFSE sequence, T2-weighted images (T2WI). (a) Wide hemispheric fissure, and “viking helmet” appearance of the lateral ventricles on a coronal image. (b) “Tear drop” configuration (colpocephaly) as a result of enlargement of occipital horns of the lateral ventricles in the axial projection. (c) “Sunray” appearance of brain sulci in the midsagittal plane.

The characteristic appearance of callosal agenesis makes this anomaly easily and early recognizable on prenatal ultrasound and MRI: wide interhemispheric fissure (Figure 1(a)), upward bulging of the 3rd ventricle, parallel lateral ventricles away from the midline—racing car sign (Figure 1(b)), widening of the atria and occipital horns of the lateral ventricles (colpocephaly)—“tear drop” configuration on axial scans (Figure 1(b)), moose head or viking helmet appearance of the frontal horns (Figure 1(a)), and the sulci on the medial aspect of the hemispheres converging towards the 3rd ventricle due to lack of cingulate gyrus—sunray appearance (Figure 1(c)).

MRI allows for visualization of the bundles of Probst—evidence that callosal fibers are not really agenetic but heterotopic, lying parasagittally on both sides and giving the lateral verticle appearance of moose head or viking helmet on coronal images.

In contrast to patients after callosotomy, individuals with callosal agenesis show only weak evidence of a “disconnection syndrome” which suggests that brain plasticity allows for forming alternative pathways of interhemispheric transfer in cases of this congenital anomaly [1].

One has to remember that as the three interhemispheric commissures develop together, callosal agenesis is only rarely isolated: it is accompanied by hippocampal commissure agenesis and in 50% of cases also by anterior commissure agenesis or hypoplasia [5].

**Figure 2:**
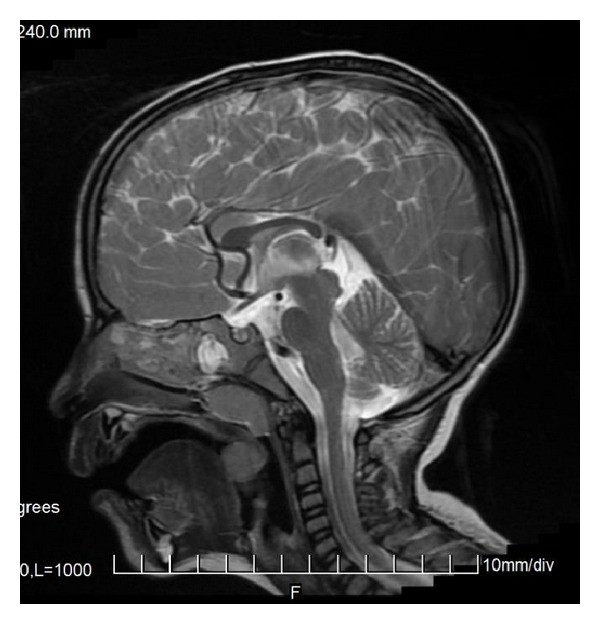
FSE sequence, T2WI, sagittal plane. Partial callosal agenesis in the form of its shortening.

There are various forms of partial callosal agenesis. In the most frequent form the corpus callosum is simply shortened (Figure 2).

**Figure 3:**
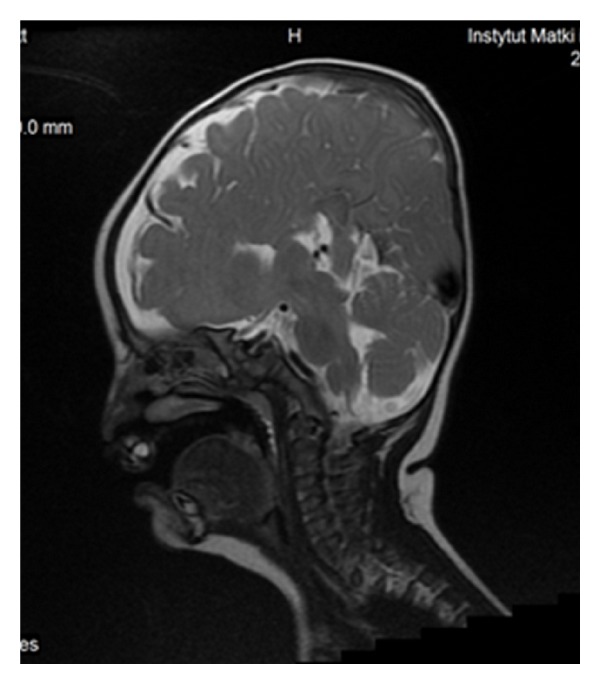
FSE, T2WI, sagittal plane. Rudimentary unmyelinated callosal splenium in a 6-month-old boy with semilobar holoprosencephaly.

**Figure 4:**
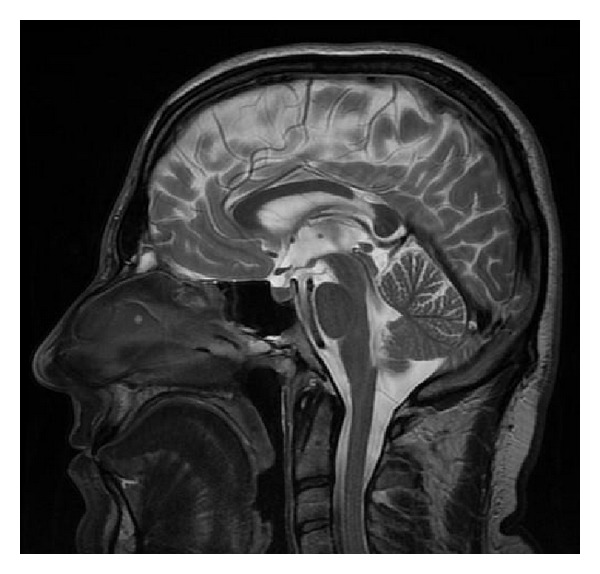
FSE, T2WI, sagittal plane. Two separate parts of the corpus callosum in a 47-year-old man—an incidental finding.

**Figure 5:**
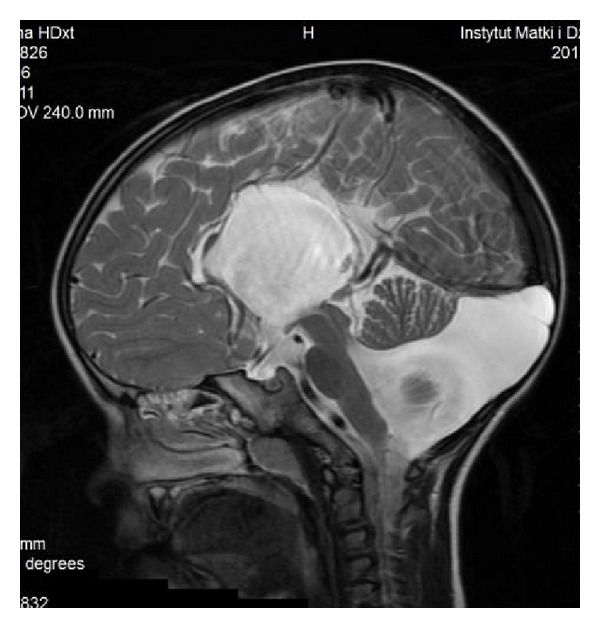
FSE, T2WI, sagittal plane. Severe callosal hypoplasia in a 4-year-old boy with macrocephaly and the Dandy-Walker syndrome. Rudimentary anterior portion of CC.

Less frequently one can observe only a rudimentary part of the corpus callosum (genu or splenium—Figure 3) or two separate parts (anterior and posterior) (Figure 4). Various degrees of callosal hypoplasia may be seen (Figure 5).

**Figure 6:**
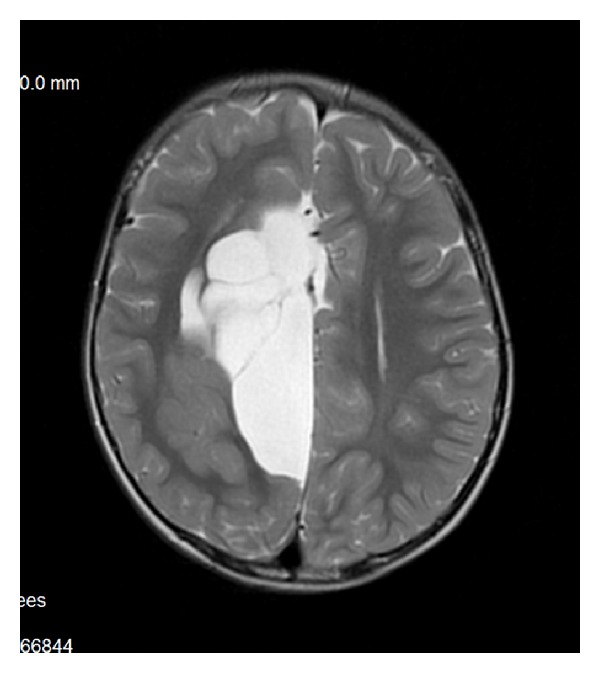
FSE, T2WI, axial plane. Agenesis of the corpus callosum with multilocular interhemispheric cyst and cortical heterotopia on the right side of the brain.

**Figure 7:**
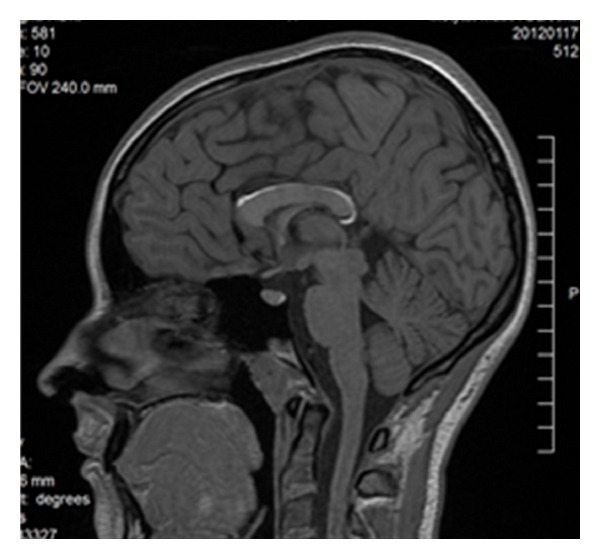
SE sequence, T1WI, sagittal plane. Dorsal tubulonodular lipoma overlying the thick and shortened CC.

**Figure 8:**
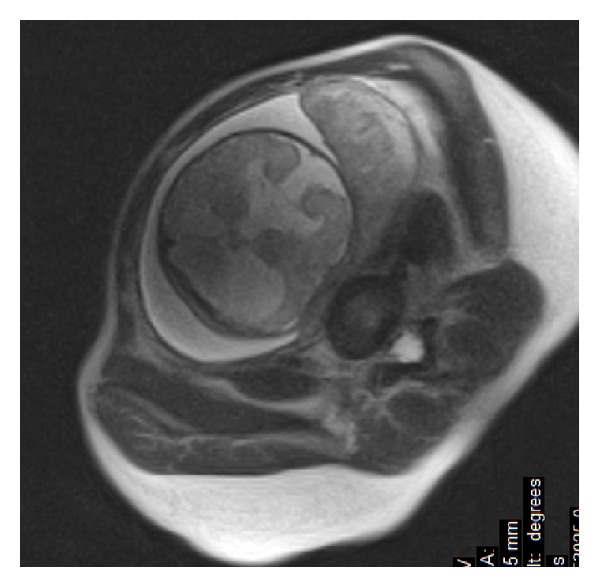
Fetal MRI. SSFSE, T2WI, coronal plane. Agenesis of the septum pellucidum and of the corpus callosum.

Suspected defects of the corpus callosum should be confirmed by MRI because in 80% of cases they coexist with other CNS pathologies. Interhemispheric cyst is one of them. It may be communicating (upward bulging of the ventricular tela choroidea or a single cyst) or noncommunicating, the latter resulting from midline meningeal dysplasia. The non-communicating cysts are usually multilocular and associated with malformations of cortical development (Figure 6). Interhemispheric meningeal lipoma is also a form of midline meningeal dysplasia and may accompany congenital callosal anomalies (Figure 7) although dorsal tubulonodular lipoma can also be found in people with normal corpus callosum. Callosal agenesis may be associated with septal agenesis (Figure 8). Callosal abnormalities are found in a great number of other brain malformations, for example, Chiari II malformation, holoprosencephaly (Figure 3), Dandy-Walker syndrome (Figure 5), PHACE syndrome (posterior fossa anomalies, hemangioma, arterial lesions, cardiac abnormalities/aortic coarctation, eye abnormalities), and microcephaly [5]. For example the authors found the increased frequency of callosal abnormalities in cases of the Nijmegen breakage syndrome in which microcephaly is a hallmark of the disease [10, 11].

Isolated callosal anomalies are often asymptomatic and may remain undetected unless highly specialized neuropsychological tests are performed [9].

**Figure 9:**
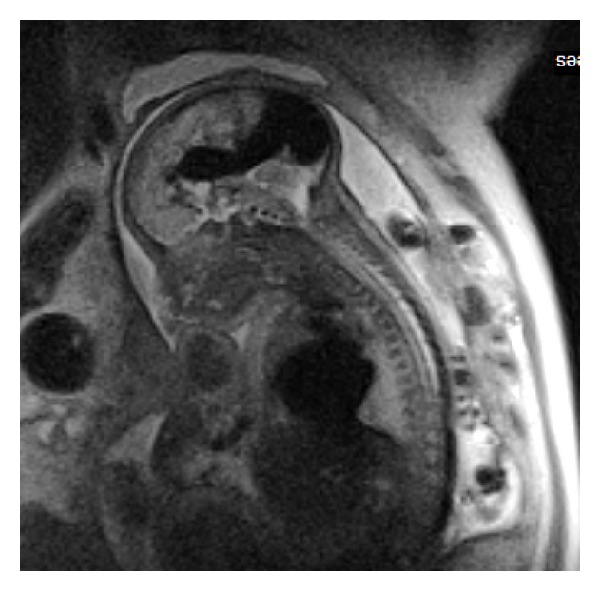
Fetal MRI—23rd week of gestation. SSFSE, T2WI, sagittal plane. Vein of Galen malformation (VOGM) causing callosal hypoplasia.

Underdevelopment of the corpus callosum may be caused by other congenital abnormalities which do not allow for its normal development. In our material there is a case of vein of Galen malformation diagnosed at the 23rd week of gestation, which resulted in callosal hypoplasia (Figure 9) [12].

### 3.2. Phakomatoses

**Figure 10:**
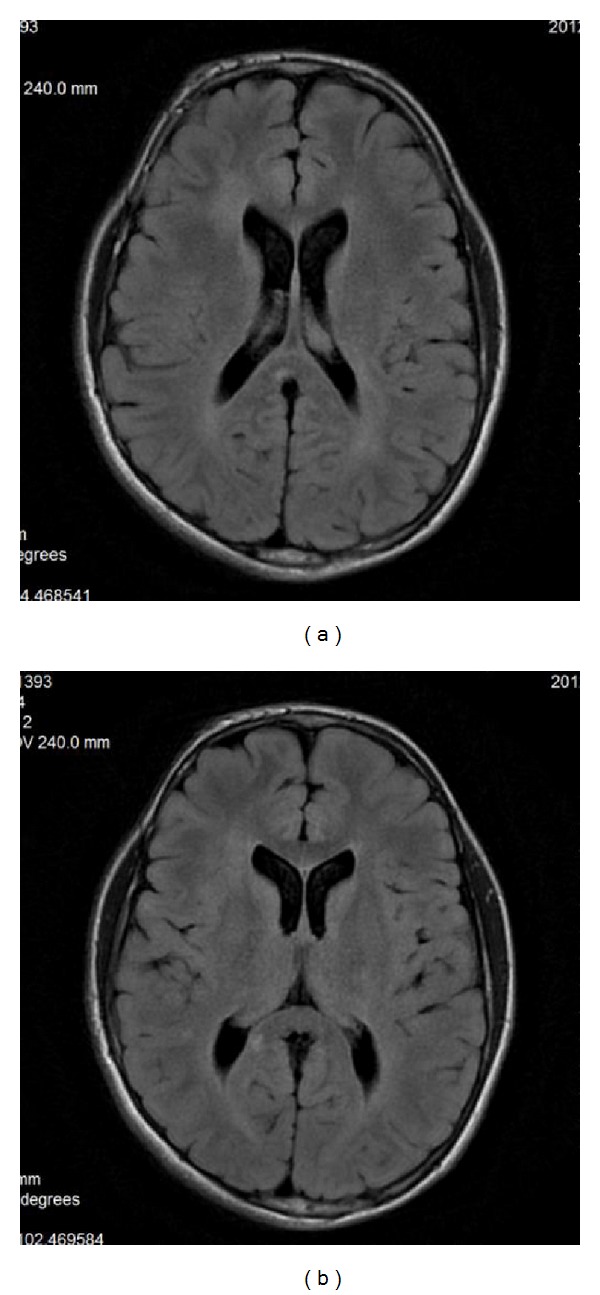
(a), (b) FLAIR sequence, axial plane. 12-year-old boy with NF1. Two hyperintense lesions in the callosal splenium—they were absent on the previous examination at the age of 10.

Phakomatoses belong to congenital diseases in which callosal abnormalities are observed. Neurofibromatosis type 1 or von Recklinghausen disease is the most frequent of them, with the estimated incidence of 1 : 3000. Neurofibromatosis bright objects (UNO), called formerly unidentified bright objects (UBO), are T2 hyperintense and appear most often in the basal ganglia, brainstem, and posterior fossa. They are also found in the corpus callosum, mainly in the splenium (Figures 10(a) and 10(b)). UNO are rare before the age of 4 years; they increase in number and volume till the age of 10–12 years and tend to resolve thereafter, so that after the age of 20 they are almost never seen. Usually they do not undergo malignant transformation but they can, so follow-up MRI studies are very important in NF1 patients [13]. Besides it has been shown that NF 1 children have a significantly larger corpus callosum while their IQ is significantly lower than in control subjects [14]. Enlargement of the rostral body, anterior and posterior midbody of the corpus callosum in these patients seems to be correlated with impairments in academic or visuospatial skills and motor coordination but may facilitate attention [1].

**Figure 11:**
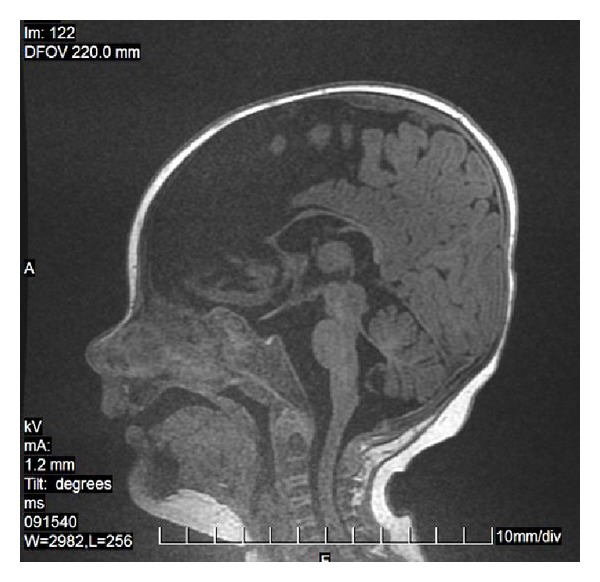
FSPGR sequence, 3D/T1WI, sagittal plane. Callosal hypoplasia in the Bloch-Sulzberger syndrome. Partially empty sella is additionally seen.

Higher incidence of callosal agenesis/dysgenesis is described in other neurocutaneous syndromes, among them are the Sturge-Weber syndrome, tuberous sclerosis complex, and Bloch-Sulzberger syndrome (Figure 11) [15].

## 4. Inborn Neurometabolic Diseases

### 4.1. X-Linked Adrenoleukodystrophy (X-ALD)

**Figure 12:**
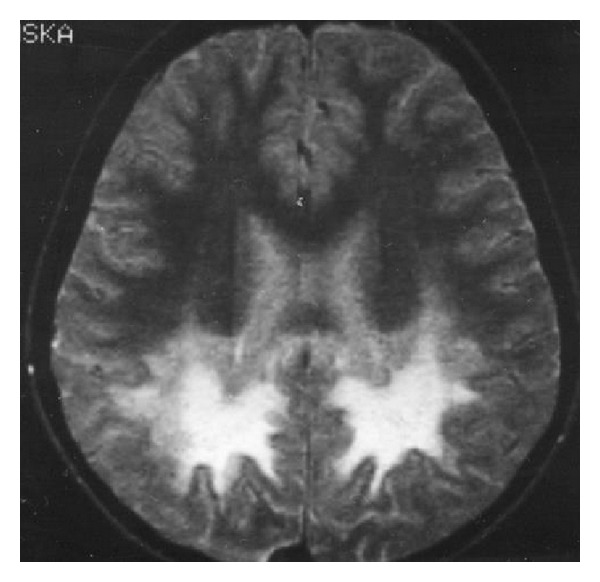
SE, T2WI, axial plane. Typical pattern of X-ALD with involvement of the callosal splenium and occipital and parietal lobes.

X-ALD is an inborn disorder of peroxisomal fatty acid beta oxidation which results in the accumulation of very long chain fatty acids in tissues. It affects mainly the myelin in the central nervous system, the adrenal cortex, and the Leydig cells in the testes. In the most typical form which accounts for approximately 80% of the cases demyelination involves callosal splenium and spreads symmetrically into occipital and parietal lobes (Figure 12) and then forward. In about 20% of patients the disease begins in the callosal genu and frontal lobes and spreads backward [16]. Contrast enhancement of the zone of active demyelination is usually observed which is uncommon in neurometabolic diseases and therefore characteristic of this disease.

## 5. Others

**Figure 13:**
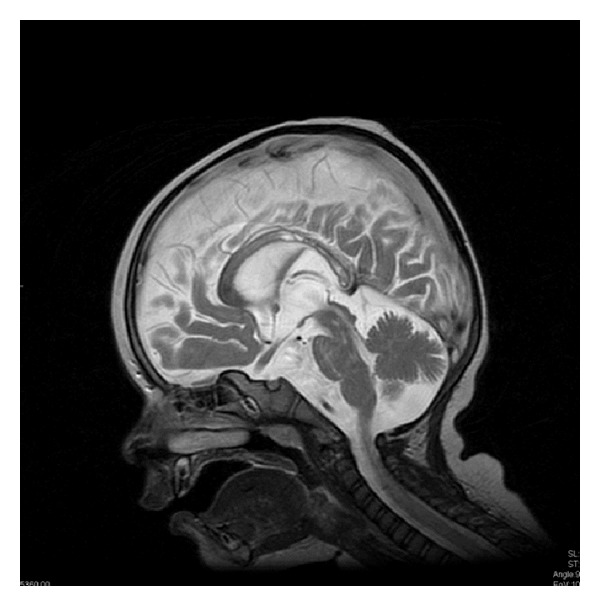
FSE, T2WI, sagittal plane. 13-month-old boy with the Krabbe disease. Diffuse demyelination of the corpus callosum with relative sparing of its ventral and dorsal borders. Six months earlier the corpus callosum was intact.

**Figure 14:**
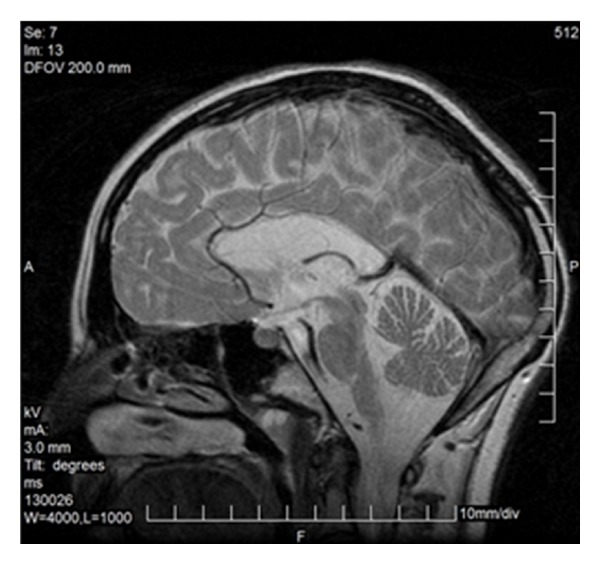
FSE, T2WI, sagittal plane. Leukoencephalopathy with vanishing white matter—corpus callosum is present but demyelinated to such an extent that practically indistinguishable from the cerebrospinal fluid on T2-weighted images.

**Figure 15:**
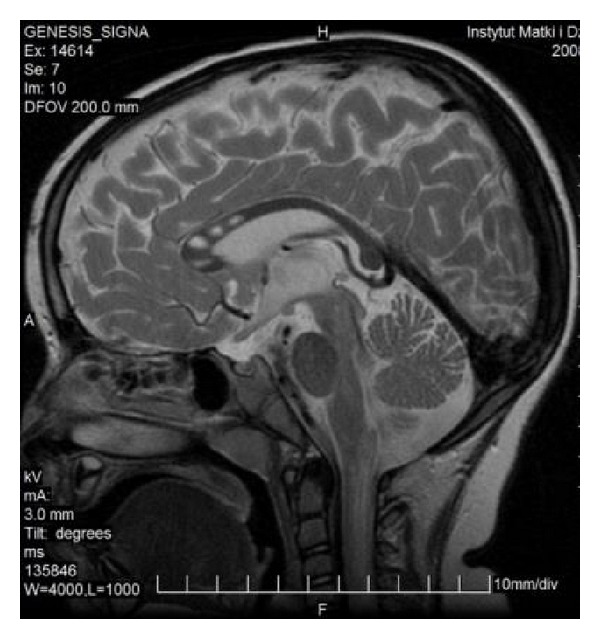
FSE, T2WI, sagittal plane. Four year-old boy with a mitochondrial disease, most likely MERFF. The lesions in the anterior part of the corpus callosum are progressive; 1.5 years earlier there was only a trace of T2 hyperintensity in the callosal genu.

The anterior part of the corpus callosum is involved in the Alexander disease. Callosal demyelination is observed in many neurometabolic diseases, for example, in globoid leukodystrophy (Krabbe disease) (Figure 13), metachromatic leukodystrophy, leukoencephalopathy with vanishing white matter (Figure 14), and mitochondrial diseases (Figure 15). Lack of myelination of the corpus callosum is an element of the Pelizaeus-Merzbacher disease.

In the course of neurometabolic diseases callosal agenesis is also observed, among others in nonketotic hyperglycinemia, Menkes kinky hair disease, Hurler disease or maple syrup urine disease [17]. In other diseases from this group secondary changes occur within the corpus callosum. The example is phenylketonuria in which loss of volume and shape abnormalities are observed in the corpus callosum [18].

## 6. Acquired Demyelinating Diseases

### 6.1. Multiple Sclerosis (MS)

**Figure 16:**
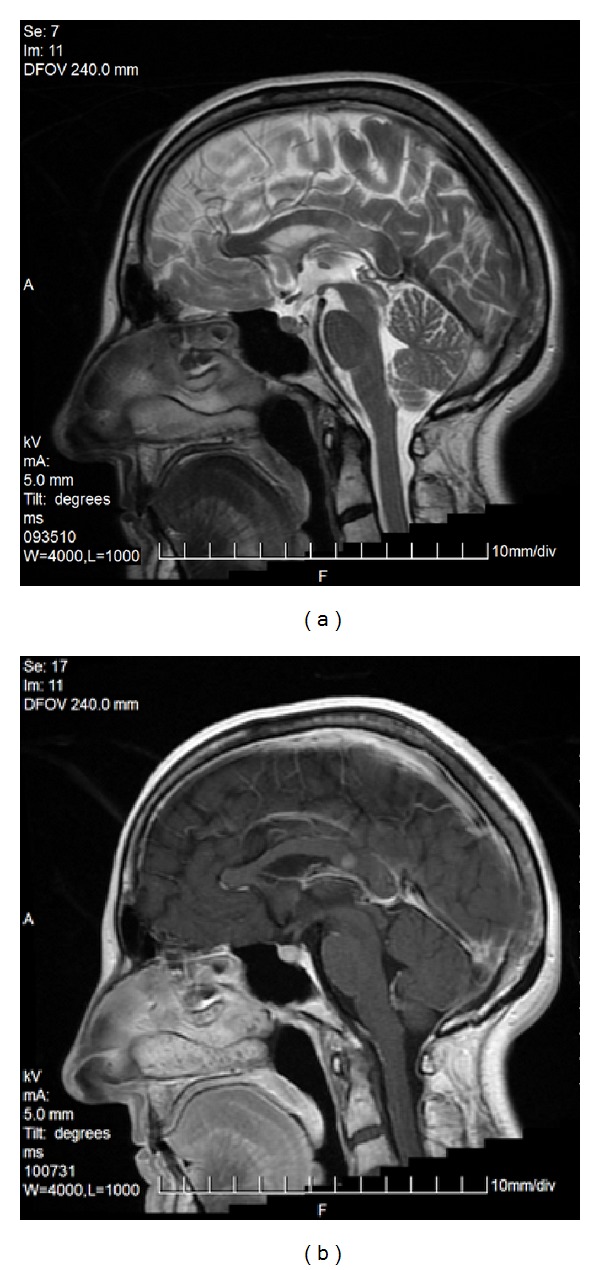
Multiple sclerosis: plaque in the isthmus of the corpus callosum (FSE, T2WI, sagittal plane (a)) with contrast enhancement after gadolinium administration (SE, T1WI after gadolinium administration, sagittal plane (b)).

Callosal involvement is typical of MS although it has never been included in the evolving diagnostic criteria of this disease [19]. The typical locations of demyelinating lesions in the course of MS are periventricular, juxtacortical, infratentorial, or spinal cord. So callosal lesions should be regarded as periventricular. In the acute phase of demyelination the plaques demonstrate contrast enhancement (Figures 16(a) and 16(b)), increased diffusion-weighted imaging (DWI) signals, and increased apparent diffusion coefficient (ADC) [20]. Cognitive impairment in benign MS has been shown to be associated with the extent of corpus callosum damage [21].

### 6.2. Marchiafava-Bignami Disease

The Marchiafava-Bignami disease is characterized by callosal demyelination and necrosis with subsequent atrophy. It is classically associated with chronic alcoholism but it has also been described in patients with malignancy and nutritional deficiencies. The lesions are T2- and FLAIR hyperintense which reflects edema and myelin damage. Necrosis in the chronic stage is reflected by T1-hypointensity but lesions may also regress [22].

## 7. Infection and Inflammation

### 7.1. Streptococcus Meningitis

**Figure 17:**
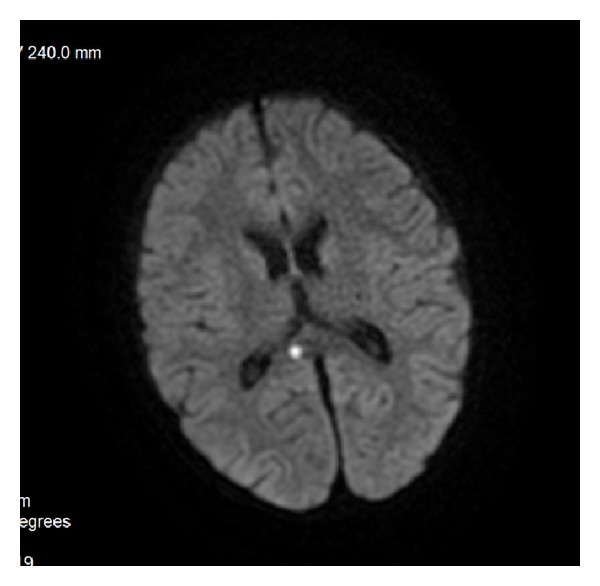
DWI sequence, axial plane. Focal infarct of the callosal splenium as a result of vasculitis in the course of streptococcal meningitis.

Cerebrovascular involvement is common in group B streptococcus meningitis, especially in neonates, but also in older children. There are two main patterns of brain infarction: deep perforator arterial stroke to basal ganglia, thalamus, and periventricular white matter and focal cortical infarctions [23]. In our archive there is a case of this disease with the only focus in the callosal splenium (Figure 17). In this case, in contrast to the generally poor prognosis with severe disability or death, the outcome was good.

### 7.2. The *Lyme* Disease

**Figure 18:**
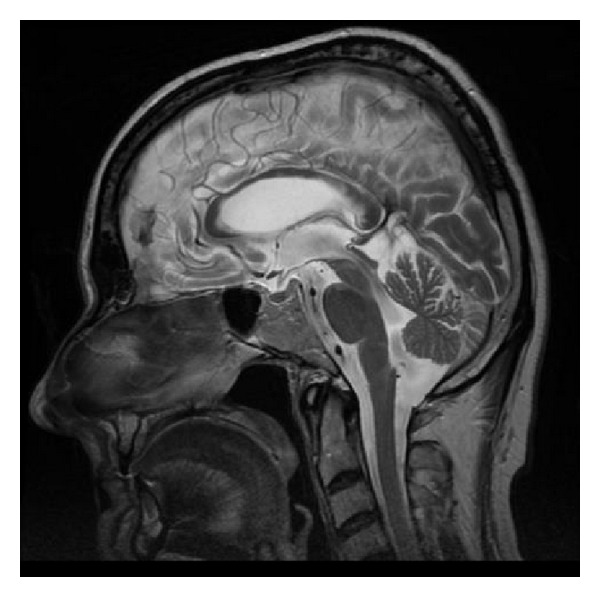
FSE, T2WI, sagittal plane. Callosal involvement in borreliosis.

The *Lyme disease*, caused by Borrelia burgdorferi, belongs to infectious diseases that are most commonly mistaken for MS [24]. FLAIR and T2-hyperintense foci may be seen in the same localization which is typical of MS, including the corpus callosum (Figure 18). MRI alone is often misleading and the presence of anti-*B. burgdorferi* antibodies in the plasma or cerebrospinal liquid is an indication for antimicrobial treatment.

### 7.3. Subacute Sclerosing Panencephalitis (SSPE)

**Figure 19:**
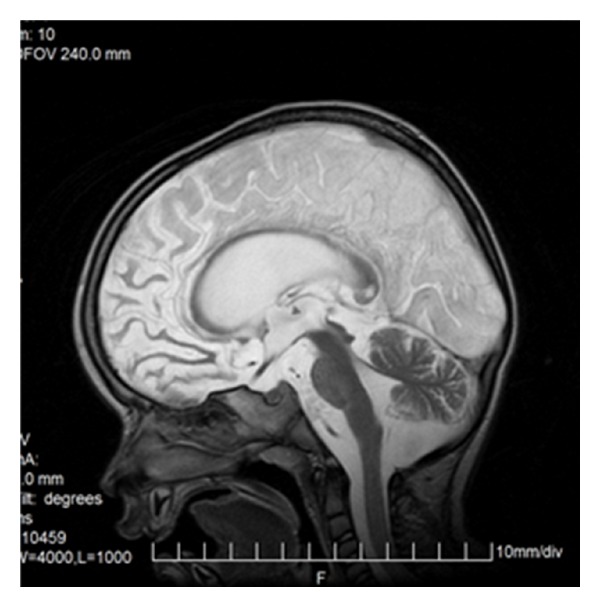
FSE, T2WI, sagittal plane. Callosal involvement in subacute sclerosing panencephalitis.

SSPE is a progressive disease considered to be caused by persistent measles virus. In typical setting the lesions in the white matter are bilateral, asymmetric, and T2-hyperintense and involve the parietal and temporal lobes in the acute stage. As the disease progresses lesions become more prominent, and periventricular white matter, corpus callosum, and basal ganglia can be involved (Figure 19) [25].

## 8. Lesions of Vascular Origin

### 8.1. Ischemic

**Figure 20:**
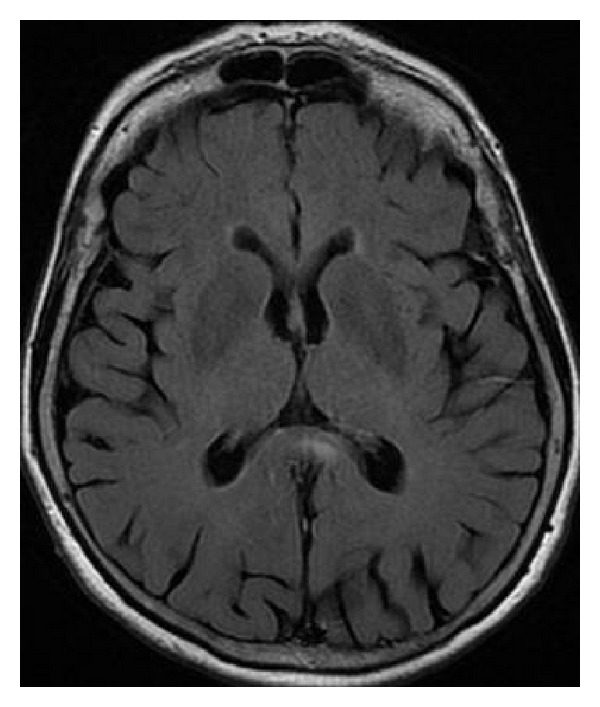
FLAIR, axial plane. Old isolated infarct in the callosal splenium.

**Figure 21:**
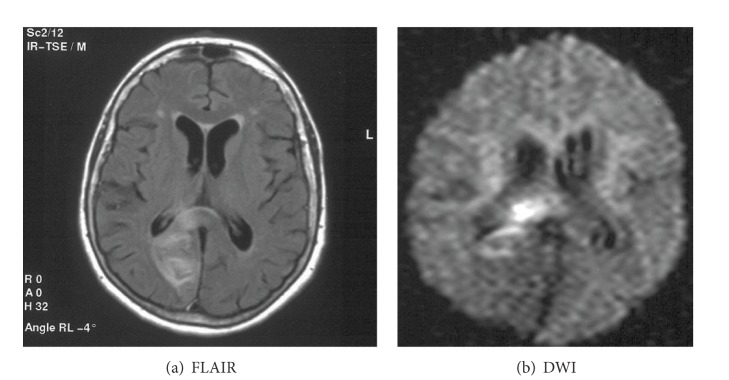
Acute stroke of the right occipital lobe involving callosal splenium as well.

Corpus callosum has rich blood supply from the anterior communicating artery (via the subcallosal and medial callosal arteries which deliver blood to the anterior part of the corpus callosum), the pericallosal artery which supplies the body, and the posterior pericallosal artery, a branch of the posterior cerebral artery, which feeds the splenium. Isolated callosal infarcts are therefore uncommon. If present, they affect callosal splenium more often than the body and genu (Figure 20) [26]. They rather accompany larger territorial infarcts (Figures 21(a) and 21(b)).

### 8.2. Vascular Malformations

Arteriovenous malformations of the corpus callosum account for 9–11% of all cerebral AVMs [7]. They are often asymptomatic and diagnosed in patients with intracranial, most frequently intraventricular, hemorrhages. The MRI pattern is typical with flow voids in the corpus callosum.

## 9. Tumors

### 9.1. Glioblastoma Multiforme

Glioblastoma multiforme (GBM) (WHO grade IV) is the most common and most aggressive malignant primary brain tumor. Callosal GBM, in addition to the corpus callosum, affects also both cerebral hemispheres resulting in the typical “butterfly glioma” appearance with solid intense contrast enhancement in the corpus callosum [27].

### 9.2. Gliomatosis Cerebri

**Figure 22:**
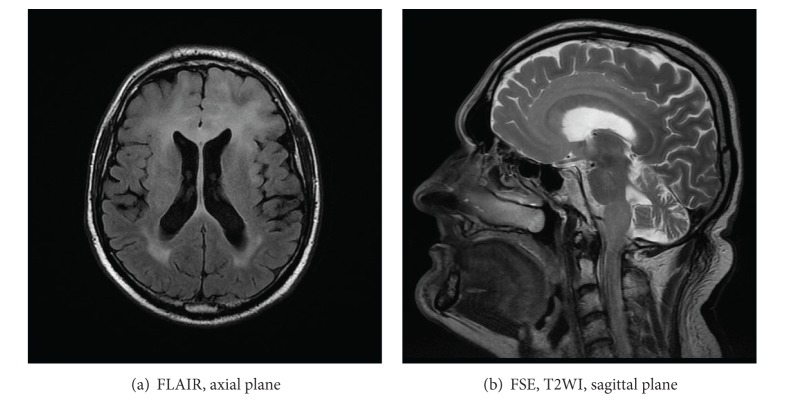
Gliomatosis cerebri.

Gliomatosis cerebri, WHO grade III, does not form a solid tumor but diffusely infiltrates the brain tissue. The architecture of the brain is preserved but the affected portions of the brain are swollen. Loss of distinction between grey and white matter is observed. Usually bilateral widespread invasion with involvement of the corpus callosum is found (Figures 22(a) and 22(b)) [28]. In 80% of cases callosal genu is affected, in 60% the body, and in 40% the splenium. The lesions are T2-hyperintense; on T1-weighted images they display isointense or—rarer—hypointense signal intensity. Mass effect and contrast enhancement are minimal [29].

### 9.3. Oligoastrocytoma

**Figure 23:**
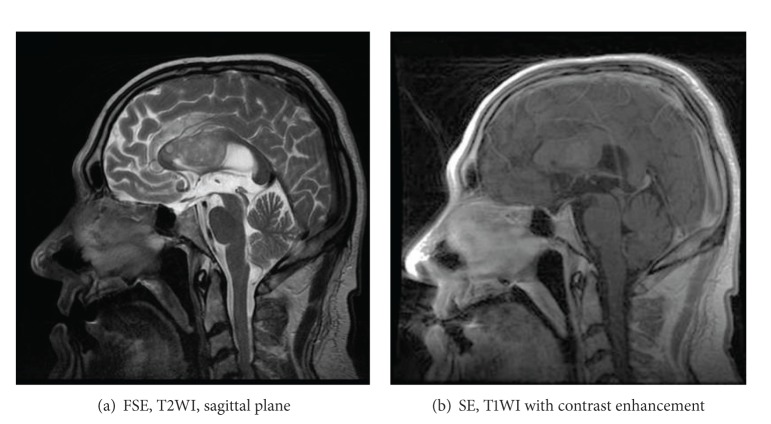
FSE, T2WI, sagittal plane. 51-year-old man with a biopsy-proven oligoastrocytoma G2.

Oligoastrocytoma (mixed glioma) occurs in two main types: well-differentiated oligodendroglioma (WHO grade II) and its anaplastic variant (WHO grade III). The most frequent locations are the frontal lobes and these tumors may involve the corpus callosum and extend through it to the contralateral hemisphere producing a “butterfly glioma” pattern (Figures 23(a) and 23(b)). Signal intensity may be mixed due to cystic elements and calcifications; “dot-like” contrast enhancement is often seen although many tumors do not enhance [30].

### 9.4. Lymphoma

**Figure 24:**
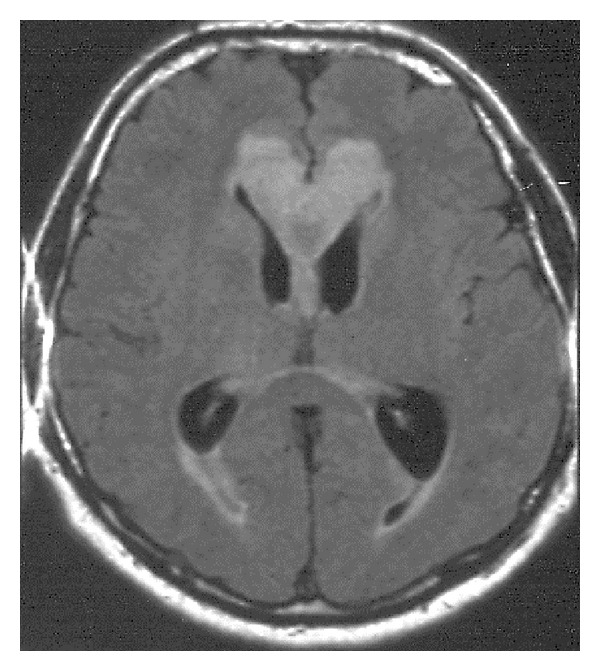
FLAIR, axial plane. Lymphoma affecting callosal genu.

Primary CNS lymphoma accounts for approximately 16% of primary brain tumors. Most of them are non-Hodgkin's and represent B-cell type. They are most often isointense-hypointense on T1-weighted images, hypointense on T2-weighted images and enhance homogeneously with gadolinium-based contrast media. In classic presentation the tumors involve the corpus callosum in a butterfly pattern (Figure 24). In patients with immunodeficiency lymphoma is more often multifocal, irregular, and heterogeneous in terms of signal intensity and ring enhancing [31].

### 9.5. Metastases

**Figure 25:**
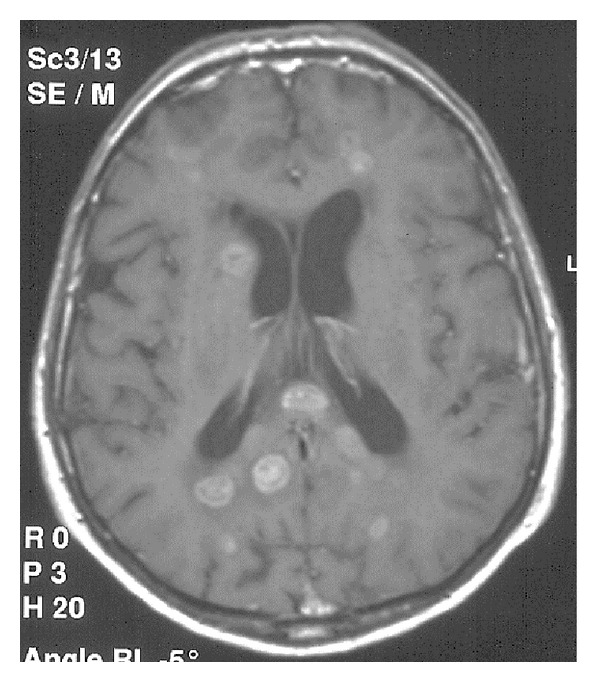
SE, T1WI after gadolinium administration. Lung cancer metastases to the corpus callosum and both cerebral hemispheres.

Corpus callosum may also be affected by metastases although it is reported to be rare. Callosal involvement is more frequent in case of infiltration by a lesion from the adjacent structures [8]. In our material there is a case of metastases of the lung cancer directly to the corpus callosum (Figure 25).

## 10. Traumatic and Iatrogenic Injuries

**Figure 26:**
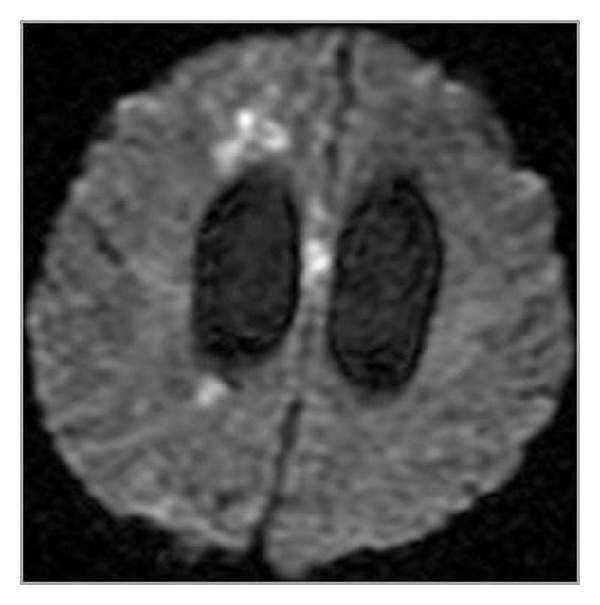
DAI—acute phase visualized on DWI.

**Figure 27:**
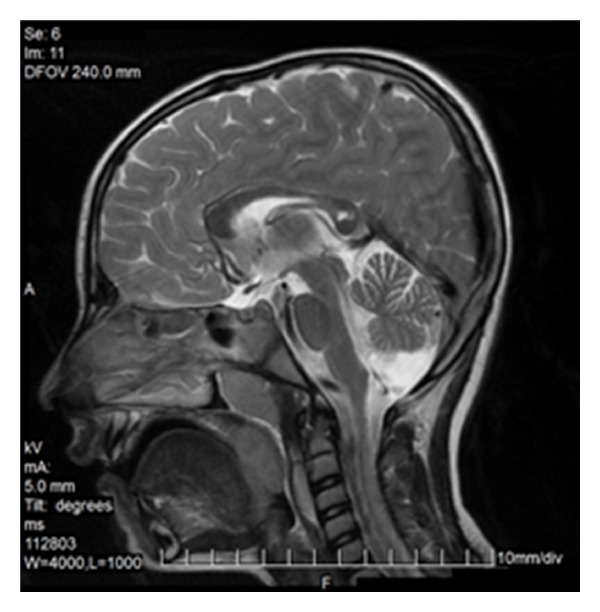
FSE, T2WI, sagittal plane. Chronic lesions in a motor vehicle accident survivor in the posterior part of the corpus callosum—localization typical of DAI.

The term “diffuse axonal injury” (DAI) refers to extensive traumatic lesions in white matter tracts. This kind of injury is the result of traumatic shearing forces that occur when the head is rapidly accelerated, decelerated, or rotated. Motor vehicle accidents are the most frequent cause of DAI but it can also be a result of child abuse, for example, in shaken baby syndrome. Corpus callosum belongs to the most frequently injured parts of white matter. The splenium and the undersurface of the posterior body are mainly involved due to vicinity of the falx cerebri. The lesions are typically small (1–15 mm) and invisible on CT. MRI is a method of choice in their diagnosis. They are T2-hyperintense but first of all show diffusion restriction with reduced ADC values (Figure 26) [32, 33]. Chronic lesions are seen as posttraumatic scars in the survivors (Figure 27). 

**Figure 28:**
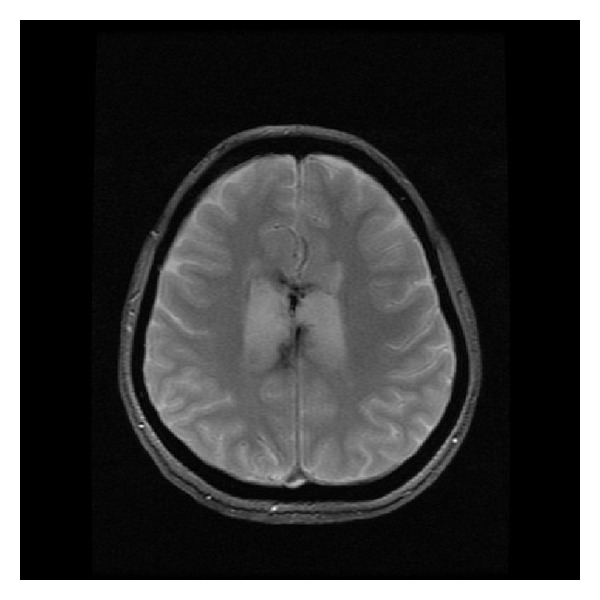
GRE sequence, T2∗WI, axial plane. Hemosiderin deposits in the corpus callosum.

**Figure 29:**
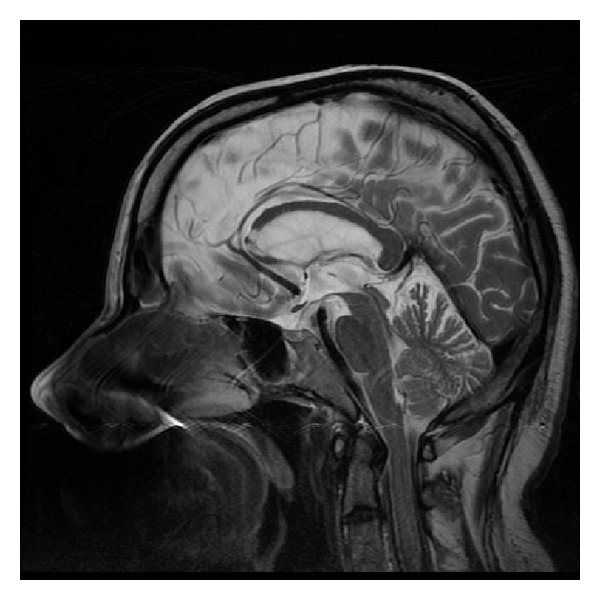
FSE, T2WI, sagittal plane. 53-year-old man with epilepsy, after head trauma. Callosal genu is torn.

 After hemorrhagic injury hemosiderin deposits may be seen in the corpus callosum (Figure 28). It may be also torn, as in Figure 29.

**Figure 30:**
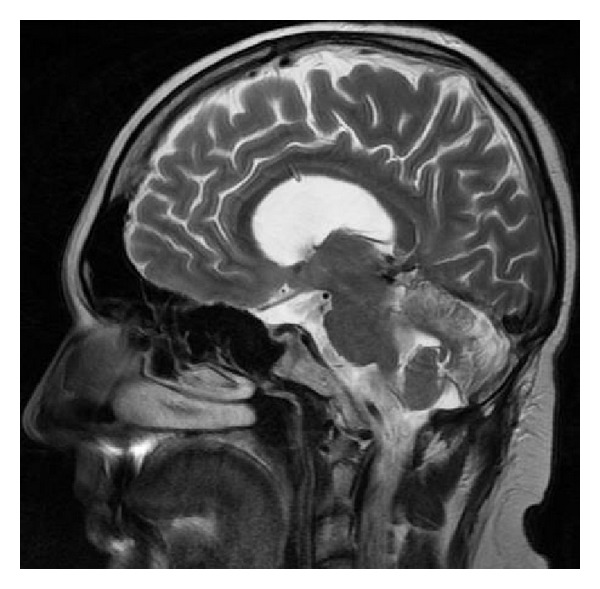
FSE, T2WI, sagittal plane. Corpus callosum pierced by a valve.

**Figure 31:**
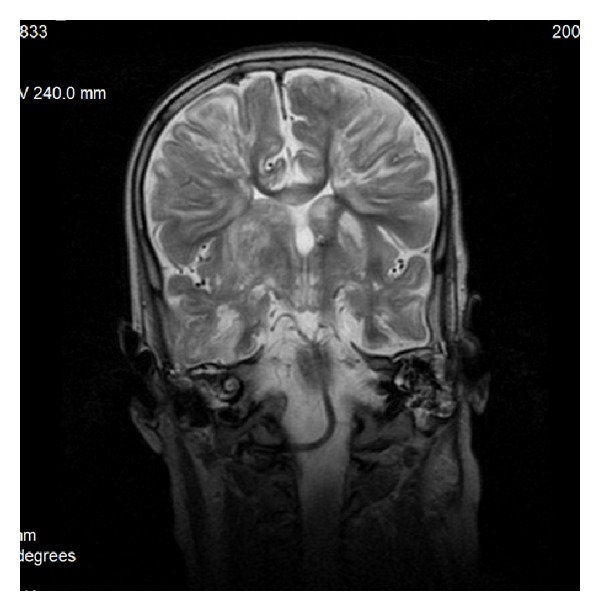
FSE, T2WI, coronal plane. Callosal injury as a result of multiple shunting procedures.

Corpus callosum may be injured as a result of shunting procedures in patients with hydrocephalus (Figures 30 and 31) [34].

**Figure 32:**
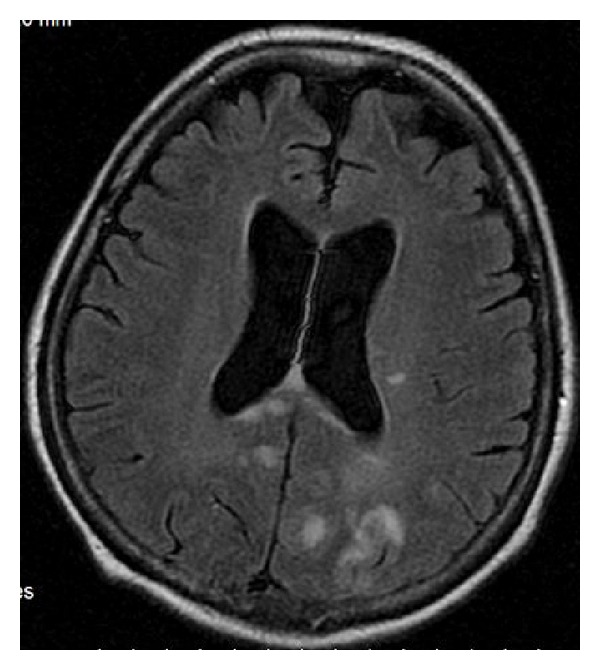
FLAIR, axial plane. Posterior reversible encephalopathy syndrome (PRES) in a 60-year-old woman with renal carcinoma and severe hypertension.

Posterior reversible encephalopathy syndrome (PRES) is a toxic-metabolic disease characterized by headache, confusion, seizures, and visual loss. It occurs in patients with malignant hypertension, eclampsia, hypercalcemia, receiving some drugs, for example, cyclosporine, after organ transplantation. That is why the condition may be considered as iatrogenic. The brain swelling is seen on MRI mainly in the posterior parts of the brain, including splenium of the corpus callosum. The symptoms tend to resolve after a period of time (Figure 32) [35].

## 11. Other/Miscellaneous

**Figure 33:**
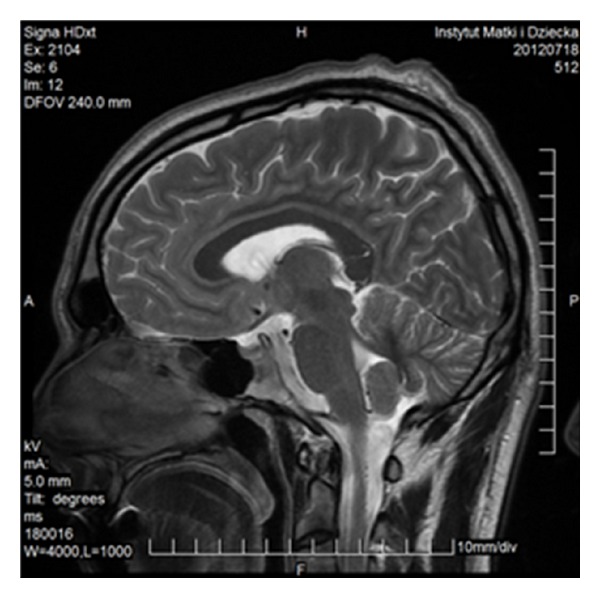
FSE, T2WI, sagittal plane. Dilated Virchow-Robin space in the callosal splenium.

Perivascular Virchow-Robin spaces may be seen in the corpus callosum as an incidental finding (Figure 33). Abnormally dilated Virchow-Robin spaces within callosum are observed in patients with mucopolysaccharidosis.

**Figure 34:**
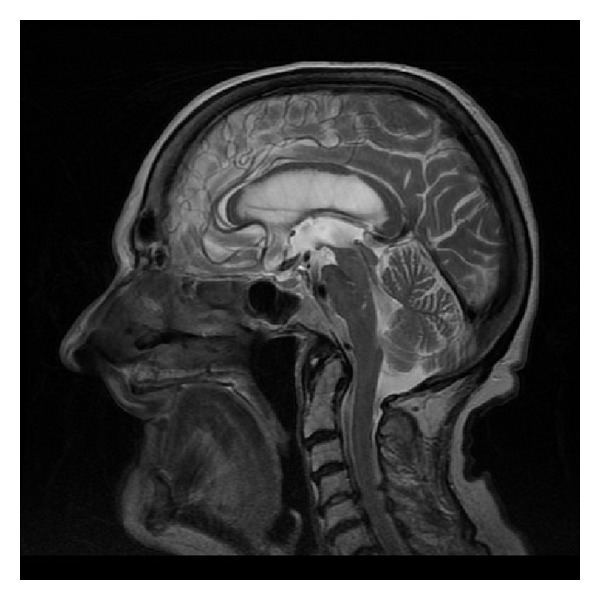
FSE, T2WI, sagittal plane. Callosal atrophy at the age of 85.

Callosal atrophy is associated with aging (Figure 34). 

In Alzheimer's disease (AD) callosal atrophy reflects loss of intracortical projecting neocortical pyramidal neurons and is more severe than in healthy subjects. The most significant atrophy in AD is noticed in callosal splenium. Callosal atrophy correlates with progression of dementia severity in AD patients [36].

**Figure 35:**
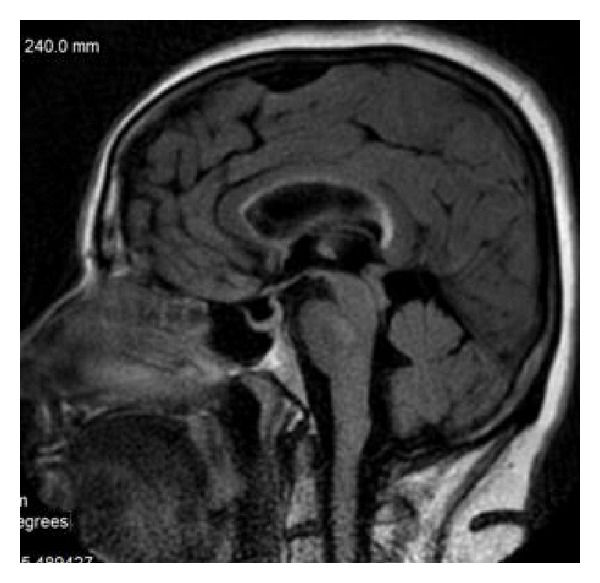
FLAIR, sagittal plane. Hyperintense band in the ventral part of CC in a 58-year-old woman with uncontrolled hypertension.

**Figure 36:**
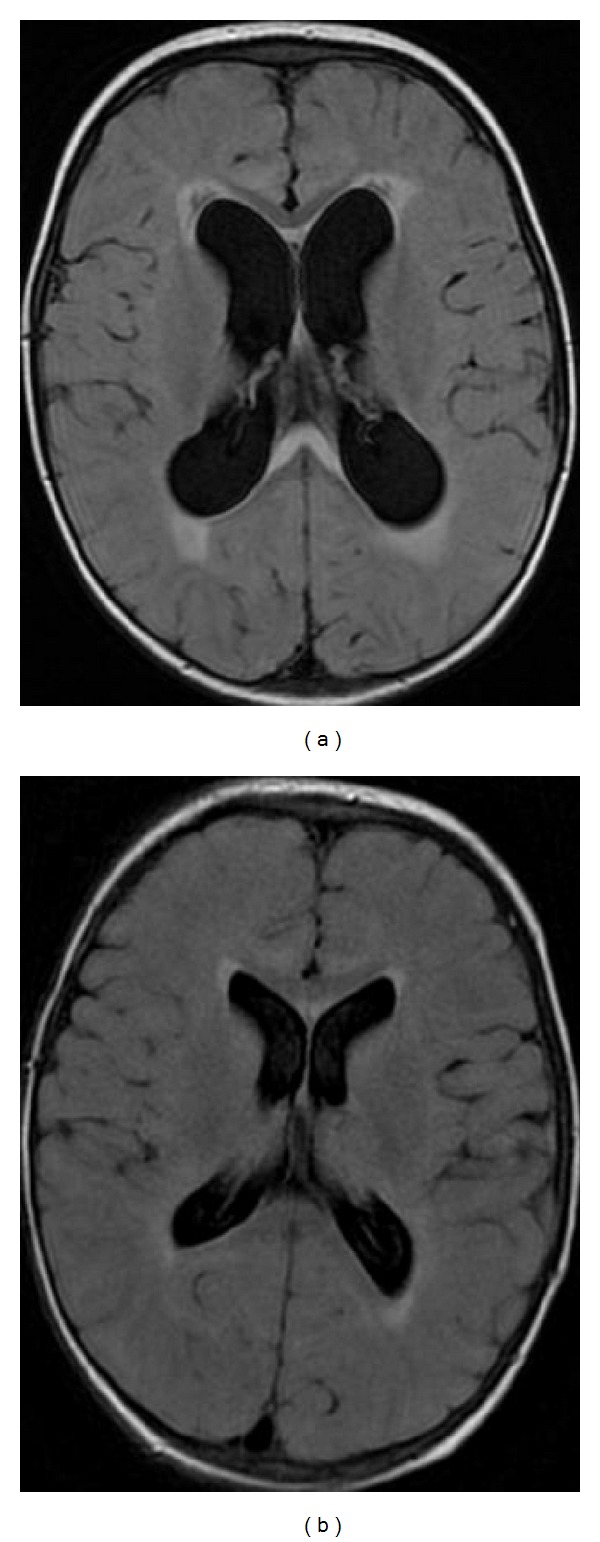
FLAIR, axial plane. (a) Five year-old boy with active hydrocephalus. (b) Three months later the ventricles are narrower and normalization of callosal signal intensity is observed.

Linear T2- and FLAIR hyperintensity of the ventral part of the corpus callosum is a frequent finding reflecting gliosis and is attributed in the literature to the elderly age, subcortical arteriosclerotic encephalopathy, and radiation therapy [8, 37]. In our experience this finding was also present in younger patients with uncontrolled hypertension and ischemic lesions in other localization, not only subcortical (Figure 35), in multiple sclerosis, PRES, and—transiently—in patients with hydrocephalus. The latter regressed after normalization of the ventricular width (Figures 36(a) and 36(b)).

**Figure 37:**
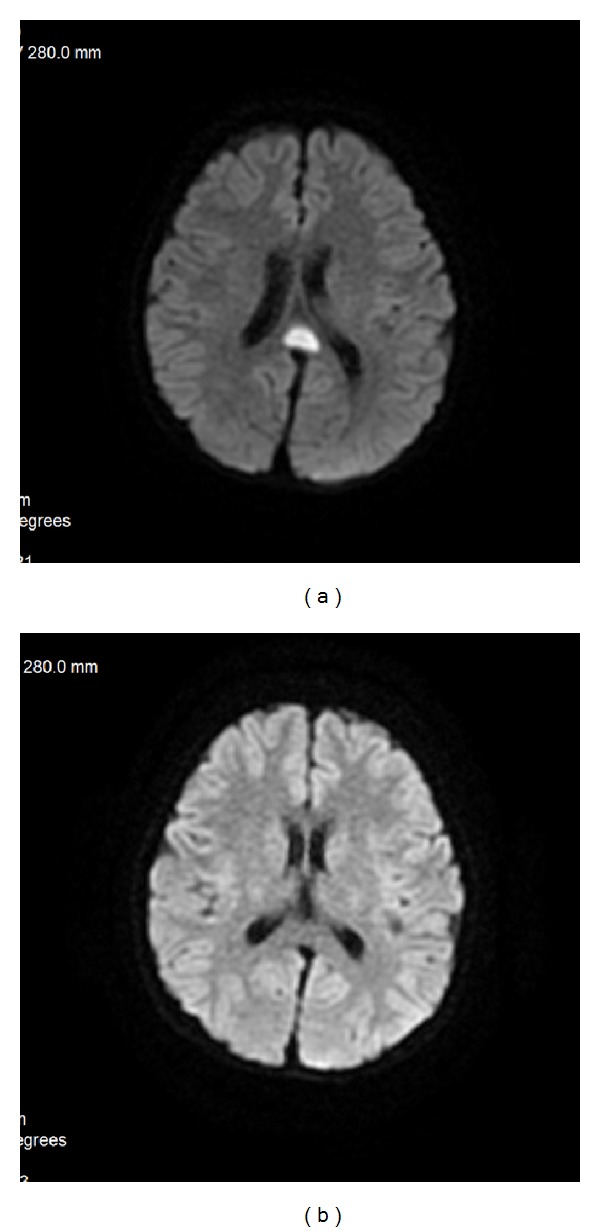
DWI sequence in axial plane. (a) Transient splenial lesion in a 9-year-old boy with school problems visualized as a hyperintense focus in the midline. (b) Six months later the lesion is absent.

Transient splenial lesion is a term attributed to the ovoid or round focus in the central part of the callosal splenium that has been described in cases of epilepsy and encephalitis. These lesions show diffusion restriction and regress with time they are regarded as intracellular (intramyelinic) edema [8]. In our material there was a case of such a transient lesion in a neurologically healthy boy referred to MRI due to “school problems” (Figures 37(a) and 37(b)).

## 12. Conclusions

Being the largest brain commissure, the corpus callosum is related to cognitive functions, social skills, problem solving, and attention. Thanks to its multiplanar nature and high tissue resolution magnetic resonance imaging is a method of choice in the assessment of the corpus callosum and its congenital and acquired pathological lesions. It is a perfect diagnosing tool from the very beginning of life, that is, from the prenatal period. Visualization of callosal involvement helps to establish diagnosis in certain disease entities.
